# Vaccination

**Published:** 1897-09

**Authors:** A. McNeil

**Affiliations:** San Francisco, Cal.


### VACCINATION.

A. McNeil, M. D., San Francisco, Cal.

Much has been said at the meetings of the I. H. A., and in representative Hahnemannian journals, that by inference, if not by innuendo, conveys the idea that a belief in vaccination and Homœopathy (there is only one Homœopathy) are antagonistic. To confute this, I quote from *The Organon* (Stratton's translation, Introduction, page 75). The same, with some difference of phraseology, is on page 226 of Dudgeon's translation.

Could vaccination protect us from the small-pox otherwise than homœopathically? Without mentioning any other traits of close resemblance which often exist between those two maladies, they have this in common—they generally appear but once during the course of a person's life; they leave behind cicatrixes equally deep; they both occasion tumefaction of the axillary glands; a fever that is analogous; an inflamed areola around each pock, and, finally, ophthalmia and convulsions.

The cow-pox would even destroy the small-pox on its first appearance; that is to say, it would cure this already existing malady, if the intensity of the small-pox did not predominate over it. To produce this effect, then, it only wants that excess of power, which, according to the law of nature, ought to *correspond* with the homœopathic resemblance, in order to effect a cure. Vaccination, considered as a homœopathic remedy, cannot, therefore, prove efficacious except when employed previous to the appearance of the small-pox, which is the strongest of the two.

In this manner it excites a disease very analogous (and consequently homœopathic) to the small-pox, after whose course the human body, which, according to custom, can only be attacked once with a disease of this nature, is henceforward protected against a similar contagion.

And in the *Leipziger Popularen Zeitscrift fur Homœopathie* for August, 1897, page 143, there is the following by Dr. H. Goullon, Weimer, Germany: "In an autographic letter of Hahnemann's, dated Coethen, August 26th, 1825, which is now before me, there are the following memorable words: 'What injury have the shameful (*schändlichen*) attacks on cow-pox vaccination done to it? Nothing! nothing at all! They have only served to cause its virtues to be more thoroughly investigated and understood.' "

I hope that hereafter those homœopaths who waste so much time in denouncing vaccination will remember these quotations from Hahnemann, particularly the last one, and those also whom they address.

